# The Endogenous Opioid System in Compulsive Eating

**DOI:** 10.3390/brainsci16070741

**Published:** 2026-07-13

**Authors:** Aneesha Janbandhu, Caden Leung, Evelyn Wu, Aidan Tom, Tobias D. Chang, Vinit Shah, Lauren Kim, Evan Robert Lauterborn, Kabirullah Lutfy

**Affiliations:** 1 Leigh High School, 5210 Leigh Ave., San Jose, CA 95124, USA; anee.ngp@gmail.com; 2 UCLA Department of Chemistry and Biochemistry, University of California, Los Angeles, 611 Charles Young Drive East, Los Angeles, CA 90095, USA; cadenleung@ucla.edu; 3 Diamond Bar High School, 21400 Pathfinder Road, Diamond Bar, CA 91765, USA; evelyn.wu.987@gmail.com; 4 Val Tech Program, Valencia High School, 500 Bradford Ave., Placentia, CA 92870, USA; aidantom11@gmail.com (A.T.); tobydc@uci.edu (T.D.C.); vinitshah307@gmail.com (V.S.); kim.laurenn01@gmail.com (L.K.); erlaut16@gmail.com (E.R.L.); 5 Department of Biotechnology and Pharmaceutical Sciences, College of Pharmacy, Western University of Health Sciences, 309 East 2nd Street, Pomona, CA 91766, USA

**Keywords:** binge-eating, obesity, food reward, opioid peptides, opioid receptors, β-endorphin, enkephalin, nociceptin, dopamine

## Abstract

**Background/Objectives:** The rates of obesity and binge-eating disorder (BED) have increased markedly over the last few decades. The onset of these conditions has been associated in part with the disruption of neural pathways that regulate food reward. Existing literature has implicated the endogenous opioid system as an important mediator of pleasure and reinforcing behaviors associated with food intake. While the relationship between opioids and food intake has been studied extensively, how dysregulated opioid signaling contributes to compulsive eating still remains unclear. Therefore, the aim of this review is to analyze the role of opioid peptides and receptors, and their interactions with dopamine in hedonic feeding. **Methods:** We conducted a narrative review of preclinical and clinical trials, incorporating studies that were relevant to opioid-mediated feeding and food reward. **Results:** β-endorphins appear to modulate the hedonic value of food, but their effects appear to be context-dependent. Enkephalins may influence motivational drive toward food, while nociceptin signaling has been linked to the preferential consumption of palatable foods under binge-like conditions. Consistent with these findings, NOP antagonism has been reported to reduce binge-like intake of a high-fat diet (HFD) without affecting homeostatic eating patterns. Lastly, chronic mu-opioid receptor (MOP) activation by palatable foods may induce neuroadaptive changes, including receptor desensitization, dopamine D2 receptor downregulation, and reward hypofunctionality, which overlap with mechanisms associated with substance use disorders. **Conclusions:** Altered MOP signaling may disrupt the hedonic and behavioral mechanisms that regulate feeding behavior. Pharmacological therapies targeting opioid and opioid-dopamine interactions may show promise for treating obesity and BED. However, additional research is still needed to clarify peptide-specific mechanisms, sex differences, and long-term neurobiological consequences associated with hedonic and compulsive eating.

## 1. Introduction

The global prevalence of obesity and binge-eating has increased markedly over the last three decades, with obesity rates surging by 27.5% in adults and 47.1% in children [1,2]. This increasing trend poses a significant public health challenge, given the association of obesity with other metabolic, cardiovascular, and psychiatric comorbidities [3]. Although genetic predisposition is a contributing factor to individual susceptibility to obesity, with studies estimating its heritability to be between 40% and 70%, food intake and dysregulated eating patterns are commonly associated with the development of these conditions [4,5]. It has been shown that palatable food consumption activates neural pathways that modulate satiety and food reward [6]. More specifically, recent studies suggest that high-fat and high-sugar diets may overstimulate hedonic neural circuits, which are involved food intake for pleasure rather than metabolic need [7,8,9]. Continuous stimulation of these hedonic pathways may be associated with excessive caloric intake and compulsive eating behaviors [10].

The endogenous opioid system, consisting of opioid peptides and receptors, regulates various hedonic and motivational processes. Endogenous opioid peptides interact with four main G-protein-coupled receptors to exert their effects: mu-opioid receptors (MOP), kappa-opioid receptors (KOP), delta-opioid receptors (DOP), and nociceptin opioid peptide receptor (NOP) [11,12]. While all four play an important role in regulating opioid signaling, MOPs have received the most attention in reward and addiction research due to their high expression in mesolimbic regions and strong association with the reinforcing properties of drugs and palatable foods. β-endorphins and enkephalins bind to MOPs with high affinity, whereas dynorphins preferentially activate KOPs [13,14]. In contrast with MOPs, KOP activation has generally been associated with promoting aversive behaviors and dysphoria, functioning as a mechanism that counteracts the hedonic effects mediated by MOPs [15,16,17]. DOPs, primarily activated by enkephalins, are important mediators of mood and analgesia, but appear to be less central to reward processing [18]. In addition to the classical opioid receptors, NOP, which is exclusively activated by nociceptin, has emerged as another potential regulator of feeding-related behaviors. Although NOPs have not gained much attention in the context of food addiction and reward, emerging evidence suggests that they may play a unique role in binge-eating and stress-induced feeding [19,20].

In the context of feeding, the endogenous opioid system appears to modulate the hedonic value of food through activation of mainly the MOPs [21,22]. Endogenous opioid peptides, including β-endorphins and enkephalins, act on MOPs to amplify reward signaling within the mesolimbic and cortical regions of the brain [23]. Activation of MOR signaling has also been linked with behaviors associated with pleasure [24,25]. However, continuous MOP activation has been associated with molecular neuroadaptations at the synaptic and circuit level, including but not limited to altered intracellular signaling cascades, receptor desensitization and downregulation, which may contribute to reduced inhibitory control [26,27,28,29]. These adaptations share similarities with those of substance use disorders, particularly opioid addiction.

Although the role of opioid signaling in food reward has been studied in numerous preclinical and clinical settings, a comprehensive synthesis of how dysregulated opioid signaling contributes to compulsive eating remains limited. This review consolidates current evidence on the downstream effects associated with abnormal opioid signaling in the context of palatable food consumption. This review further examines the relationship between opioid and dopamine signaling and discusses implications for potential pharmacological targets and interventions for obesity and binge-eating.

## 2. Materials and Methods

This review consolidates existing literature and studies from PubMed and Google Scholar databases, using Boolean operators (AND, OR) to combine search terms. Searches conducted combined the following terms using the aforementioned Boolean operators: MOP, MOR, KOP, KOR, DOP, DOR, NOP, ORL-1, β-endorphin, enkephalin, dynorphin, nociceptin, POMC, proenkephalin, opioid peptide, mu-opioid receptor, opioid receptor antagonist, naltrexone, naloxone, GSK1521498, food reward, binge-eating, binge-eating disorder, hedonic feeding, homeostatic feeding, compulsive eating, palatable food, high-fat diet, incentive salience, mesolimbic, nucleus accumbens, ventral tegmental area, dopamine, D1 receptor, D2 receptor, GABA, reward hypofunctionality, place conditioning, PET imaging, and obesity. The final literature search was conducted between June 2025 and April 2026, although this project initiated in early 2020s. A variety of studies were incorporated into this narrative review, including but not limited to knockout mice models, human neuroimaging and PET imaging studies, clinical trials, behavioral animal models, optogenetic studies, and place conditioning paradigm designs. In addition to primary research articles, relevant review articles and comparative studies were also included to provide context.

The inclusion criteria included peer-reviewed articles published between 1981 and 2026, written in English, that discussed opioid peptides and receptors, food reward and palatability, hedonic and homeostatic regulation, compulsive eating behaviors, and/or opioid-dopamine interactions in humans and rodents. Exclusion criteria included articles published prior to 1981, written in languages other than English, prior reviews not focusing mainly on opioids and food intake/palatability or reward as well as opioids and compulsive eating. We also excluded studies focusing solely on opioid signaling in addiction or hedonic behaviors unrelated to food reward, and studies irrelevant to the objective of the review, including but not limited to the role of opioids in obsessive–compulsive disorder. Records retrieved from both databases were compiled with duplicate studies removed. The reference lists of included articles and relevant reviews were also manually screened to identify additional eligible studies to supplement our review. In total, we screened approximately 225 publications, and 116 articles were ultimately included in this review. Due to the narrative format of the review, we acknowledge the possibility of selection bias in the inclusion and analysis of evidence presented in this paper. Thus, findings here should be interpreted with caution. Selected articles were then analyzed for relevance to the goal of the review and organized into the subsequent categories of this review. Disagreements regarding article inclusion were resolved through discussion among the authors until a consensus was achieved. All authors reviewed and agreed with the final set of included articles.

## 3. Results

### 3.1. Regulation of Feeding Behavior

#### 3.1.1. Homeostatic Feeding

Homeostatic feeding refers to the biological process by which energy intake is adjusted accordingly to meet metabolic demands and maintain long-term energy balance. This system functions to prevent energy deficiency or excess by integrating peripheral metabolic signals with central neural circuits that regulate hunger and satiety [30]. Distinct from hedonic feeding (described below), homeostatic feeding is primarily motivated by physiological needs rather than food reward and is strictly governed by coordinated endocrine and neural feedback mechanisms [31].

The hypothalamus serves as a central homeostatic regulator of food intake. The arcuate nucleus (ARC), located in the mediobasal hypothalamus, contains two antagonistic neuronal populations that regulate feeding behavior: (1) orexigenic neurons expressing neuropeptide Y (NPY) and agouti-related peptide (AgRP) and (2) anorexigenic neurons expressing proopiomelanocortin (POMC) and cocaine- and amphetamine-regulated transcript (CART). Activation of NPY/AgRP neurons stimulates appetite and feeding; activation of POMC/CART neurons suppresses food intake and enhances satiety [30,32].

**Figure 1:**
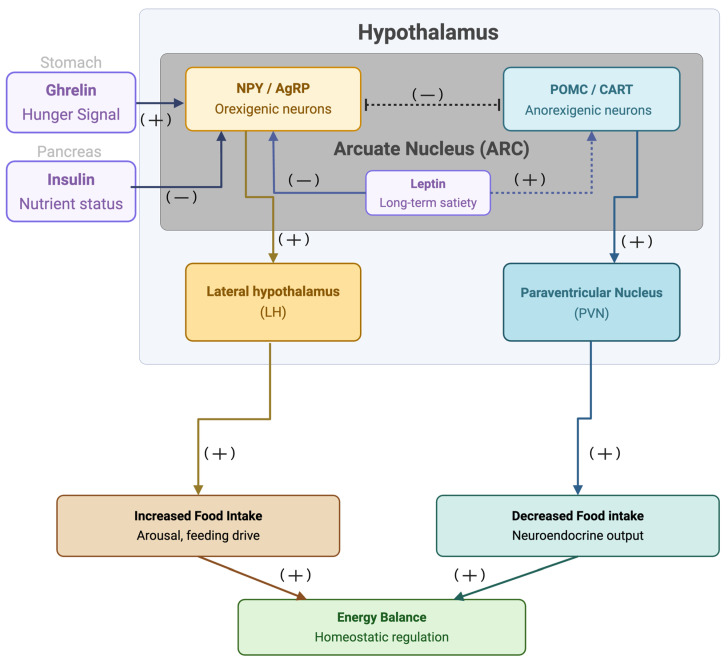
A flowchart diagram showing neural circuits regulating homeostatic feeding. The ARC of the hypothalamus contains antagonistic NPY/AgRP (orexigenic) and POMC/CART (anorexigenic) neurons that integrate peripheral metabolic hormones that are either inhibitory (i.e., leptin and insulin) or excitatory (i.e., ghrelin). Downstream projections to LH and PVN translate these signals into feeding behavior and neuroendocrine outputs that maintain homeostatic energy balance. Dashed lines indicate indirect projections. Abbreviations: ARC, arcuate nucleus; AgRP, agouti-related peptide; CART, cocaine- and amphetamine-regulated transcript; LH, lateral hypothalamus; NPY, neuropeptide Y; POMC, pro-opiomelanocortin; PVN, paraventricular nucleus.

As shown below (Figure 1), peripheral metabolic hormones also provide continuous feedback to the hypothalamic circuits in relation to the body’s energy state. Leptin acts as a signal of satiety by inhibiting NPY/AgRP neurons and activating POMC neurons [33]. Insulin exerts similar anorexigenic effects and reflects both acute nutrient availability and chronic energy balance. Ghrelin, conversely, is secreted from the stomach and acts on these neurons to stimulate hunger by activating orexigenic neurons within the ARC [34]. In addition to the ARC, other hypothalamic nuclei contribute to the integration and execution of homeostatic feeding responses through the paraventricular nucleus (PVN) and lateral hypothalamus (LH). The PVN receives projections from ARC neurons and regulates autonomic and neuroendocrine outputs that influence food intake and energy expenditure [35]. The LH coordinates arousal and initiates feeding through projections to the brainstem and forebrain regions, enabling adaptive behavioral responses to metabolic signals [36,37].

While the homeostatic feeding system is highly conserved, it is susceptible to dysregulation under conditions of chronic caloric excess. Sustained exposure to high-fat or high-sugar diets can impair sensitivity to satiety signals in the hypothalamus. This diminished sensitivity to satiety signals weakens inhibitory control over orexigenic pathways, leading to excessive food intake despite sufficient energy reserves [38,39].

#### 3.1.2. Hedonic Feeding

Hedonic feeding is characterized by food consumption driven by pleasure rather than physiological energy needs. While early models of palatability framed hedonic eating as a response to underlying nutritional deficits associated with homeostatic feeding, these models overlook the frequent overconsumption of highly palatable foods in the absence of physiological hunger. Current studies now suggest that palatability operates as a partially independent hedonic system, capable of stimulating food intake despite low homeostatic need and, in some cases, exerting its strongest effects under conditions of satiety [40,41].

The hedonic feeding framework can be distinguished into two components: (1) “liking,” the pleasurable impact of food consumption and (2) “wanting,” the motivational drive to obtain and consume. These components function through overlapping but distinct neural circuits, allowing motivation to increase independently of the pleasure derived from food. Such dissociations help explain why palatable foods may elicit robust cue-driven intake within hedonic contexts, even when subjective enjoyment does not increase proportionally [42].

**Figure 2:**
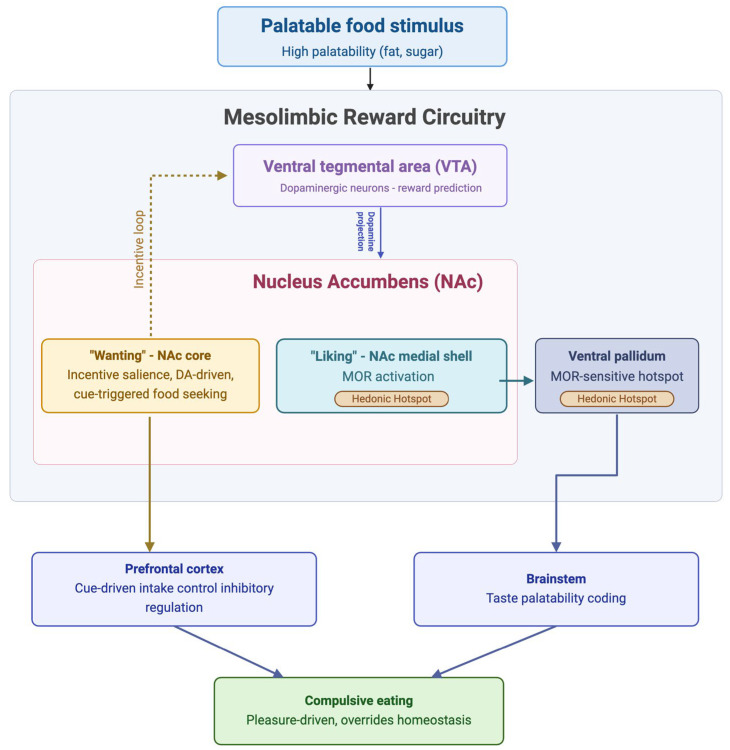
Mesolimbic circuitry governing hedonic feeding. Palatable food activates dopaminergic neurons in the VTA, projecting to the nucleus accumbens (NAc). The NAc core mediates “wanting” via dopamine-driven, cue-triggered food seeking, while the NAc medial shell and ventral pallidum act as mu-opioid receptor (MOP)-sensitive hedonic hotspots that enhance “liking.” Signals from these regions interact with the prefrontal cortex (inhibitory control) and brainstem (taste processing) to drive intake. Dysregulation of this circuit promotes compulsive, pleasure-driven eating that overrides homeostatic regulation.

The mesolimbic circuitry governs hedonic feeding (Figure 2). The various hedonic opioid-mediated effects converge within the mesolimbic reward circuitry, originating in the ventral tegmental area (VTA) and projecting to the nucleus accumbens (NAc), and connected cortical regions [43]. Within the NAc, MOP activation within the medial shell enhances palatable food intake by selectively increasing hedonic impact, while indirectly modulating dopaminergic output from the VTA to strengthen incentive motivation [44]. Hedonic hotspots, defined as the localized subregions capable of amplifying the pleasure derived from palatable tastes, have been identified in the medial shell of the NAc and the ventral pallidum, with additional contributions from the cortical and brainstem regions involved with reward processing. These hotspots are particularly sensitive to opioid signaling and modulate the “liking” aspect of food rather than metabolic needs [45]. More importantly, this circuitry overlaps substantially with pathways implicated in substance use disorders, suggesting shared neurobiological mechanisms underlying compulsive consumption [42]. As a result, hedonic eating is increasingly understood as the predictable output of neural systems designed to prioritize pleasure and reinforcement, contributing to compulsive eating behaviors.

This liking/wanting distinction is a useful organizing heuristic, but the mapping of dopamine onto “wanting” and opioid peptides onto “liking” is more sharply drawn in summary than the underlying circuitry supports. Direct electrophysiological dissociation of these components in the NAc and ventral pallidum shows a clear divergence. Intra-accumbens opioid stimulation enhances both hedonic “liking” reactions and incentive salience-related neural firing to reward-predictive cues. In contrast, dopamine stimulation in the same circuit selectively enhances incentive salience without altering hedonic impact [46]. Opioid signaling therefore contributes to both the hedonic and motivational components of food reward rather than being confined to liking alone, consistent with the enkephalin-related motivational deficits and the MOP-dependent effects on anticipatory food seeking described in Section 3.2.2 and Section 3.3.1. Dopamine, by contrast, does appear to be more consistently and selectively tied to incentive salience: across pharmacological and lesion studies, dopamine has repeatedly been found to be neither necessary nor sufficient to produce changes in hedonic liking for sensory pleasures, even though it reliably shapes cue-triggered wanting [47]. The liking/wanting framework therefore remains useful for interpreting opioid-dopamine interactions in food reward, provided it is interpreted as a difference in degree and relative specialization between the two systems rather than a strict double dissociation.

### 3.2. Endogenous Opioid System in Food Reward

#### 3.2.1. Opioid Peptides and Receptor Subtypes

Beyond its well-established role in analgesia, the endogenous opioid system plays an important part in reward processing and reinforcement. This system consists of four distinct opioid peptide families: β-endorphins, enkephalins, dynorphins, and nociceptin. Each family is derived from distinct precursor proteins and exhibits different distributions throughout the brain. Of these, β-endorphins have been most strongly implicated in food reward and hedonic feeding. The peptide is cleaved from its precursor protein proopiomelanocortin (POMC), which is predominantly expressed in neurons of the ARC [48,49]. From the ARC, β-endorphin-synthesizing neurons send projections to several nodes of the mesolimbic system that regulate food reward, including the NAc, VTA, and amygdala [50]. This neuroanatomical arrangement allows β-endorphin to directly modulate the neurocircuitry responsible for motivation and reward. Recent single-cell transcriptomic and intersectional genetic studies indicate that POMC neurons are not a uniform population but comprise molecularly and functionally distinct subpopulations. Within the ARC, POMC neurons segregate into largely non-overlapping leptin receptor-expressing and glucagon-like peptide-1 (GLP-1) receptor-expressing clusters that differ in their basic electrophysiological properties [48,51]. Other subsets are distinguished by the expression of GABAergic or glutamatergic markers, or co-expression of both, raising the possibility that individual POMC subpopulations engage distinct postsynaptic circuits despite arising from a shared precursor protein [51]. POMC neurons outside the ARC, including a population in the nucleus of the solitary tract, act on markedly different timescales than their arcuate counterparts, suppressing feeding within minutes rather than requiring sustained activation over hours [51]. This spatial and molecular heterogeneity suggests that the behavioral output of POMC activation, and by extension β-endorphin release, likely depends on which POMC subpopulation is engaged rather than reflecting a single, unified hedonic signal. Identifying which POMC subpopulations specifically project to mesolimbic reward circuitry will be an important next step for clarifying the food-reward effects discussed above.

Enkephalins, derived from proenkephalin, are more widely distributed throughout the central nervous system, found in high concentrations in the striatum, amygdala, and hypothalamus [13,52]. Unlike β-endorphin, which originates from the discrete population of ARC POMC neurons, enkephalinergic neurons are broadly expressed, allowing for more widespread modulation of reward and affective states [53]. Dynorphins, derived from prodynorphin, are localized in the hypothalamus, striatum, and hippocampus [54]. Interestingly, studies have shown that dynorphins counteract the rewarding effects of other opioid peptides and have been associated with dysphoria and aversive responses [15,16]. Different from the other three opioid peptides, nociceptin exclusively acts on NOPs. It is derived from pronociceptin, and cells expressing the precursor, especially in the central amygdala, have projections extending to ventral bed nucleus of the stria terminalis (vBNST), parabrachial nucleus (PBN), and nucleus of the solitary tract (NTS), which produce reward behavior in the context of food intake and food reward [55]. Taken together, these intricacies of the endogenous opioid system provide valuable insight into the regulation of food reward and the development of addiction-like behaviors.

#### 3.2.2. β-Endorphins, Enkephalins, and Palatable Food Intake

Early evidence for the role of β-endorphins in food reward emerges from studies examining taste-induced opioid release. Yamamoto and colleagues found that the ingestion of palatable solutions like sucrose and saccharine by Wistar rats significantly increased β-endorphin levels in both cerebrospinal fluid and plasma. In contrast, aversive solutions produced weaker and inconsistent effects. More importantly, the attenuation of sucrose-induced β-endorphin release following a glossopharyngeal nerve transection suggests that gustatory input, rather than post-ingestive metabolic signaling, is a primary trigger for the release of the endogenous opioid peptide. This frames β-endorphins as mediators of taste-driven reward rather than caloric detection, supporting their role in enhancing hedonic value and reinforcing reward-driven feeding [56].

However, when β-endorphins are examined in the context of obesity and chronic alterations of feeding, their role in contributing to overeating becomes less clear. Guinion and Peters investigated multiple models of experimental obesity and found that opioid receptor antagonism by naloxone suppressed food intake similarly across treatment groups, regardless of obesity status or hypothalamic manipulation. Furthermore, pituitary β-endorphin levels were largely unchanged by the development of obesity, except in animals rendered obese through long-term exposure to palatable diets. However, it is important to note that as a peripheral measure, pituitary β-endorphin levels do not necessarily reflect the activity within the mesolimbic reward circuitry, which is more closely implicated in hedonic feeding. Nonetheless, the lack of a generalized increase in β-endorphin levels further weakens the causal relationship between β-endorphins and obesity. Instead, they suggest that elevated β-endorphin activity may be a downstream adaptation to repeated palatable food exposure. This difference in findings is especially important to consider regarding the relationship between β-endorphins, binge-eating behaviors, and obesity [57]. However, it is worthwhile noting that mice lacking β-endorphin have been reported to have higher body weight than their wild-type controls [58]. Thus, further studies are needed to characterize the underlying mechanisms of the increased body weight in mice lacking β-endorphin and delineate the role of β-endorphin in glucose homeostasis and diet-induced obesity.

Further insight into the sensory mechanisms underlying β-endorphin activation comes from Matsumura and colleagues, who showed that dietary fat ingestion activated β-endorphin-producing neurons in the hypothalamus, but only when taste pathways remain intact. The lack of neural activation following direct gastric fat administration highlights the importance of oral sensory stimulation in engaging β-endorphin circuits. Together with Yamamoto’s findings, this suggests that β-endorphins respond preferentially to the hedonic sensory properties of food, rather than to macronutrient content alone. This has important implications for binge-eating, where consumption is often driven by highly palatable foods rich in fat or sugar rather than by homeostatic energy needs [59]. We have recently shown that mice lacking β-endorphin exhibit reduced binge-eating [60]. However, further research is needed to identify the circuit and brain regions involved in this response.

The functional specificity of β-endorphins becomes more apparent when compared with other endogenous opioids. Mendez and colleagues provide a direct comparison between β-endorphin and enkephalin signaling, revealing their distinct roles in feeding behavior. Enkephalin-deficient mice showed reduced motivation for feeding, while β-endorphin-deficient mice displayed changes in palatability-related licking behavior under conditions of deprivation. These results suggest that β-endorphins selectively enhance the hedonic value of food under conditions of increased reward, such as hunger or exposure to highly palatable foods, whereas enkephalin signaling appears to be more involved in sustaining motivational drive towards food. This differentiation within the opioid system provides a possible explanation as to why opioid antagonism can produce inconsistent effects on feeding and why binge-eating cannot be defined by a single neurochemical pathway alone [61].

Recent work by Tolentino and colleagues challenges the role of β-endorphins in binge-eating. Interestingly, mice lacking β-endorphins did not differ from controls in baseline intake, deprivation-induced feeding, or binge-like consumption. This suggests that experience-dependent processes such as learning or habit formation, along with other neurochemical systems, may override β-endorphin signaling in driving compulsive eating behaviors [62]. These findings suggest that β-endorphins may not be required for the development of binge-eating behaviors but instead function to regulate the hedonic value of food.

These findings appear inconsistent with studies reporting reduced binge-eating and impaired food reward in mice lacking β-endorphin [60]. However, rather than indicating that one set of findings is incorrect, these differences are likely to reflect variations in data analysis. In the former study by Tolentino and colleagues [62], the raw data were used. On the other hand, in the latter work by Era et al. [62], the body weight of the mice was considered and the data were converted as calorie intake per gram of body weight of the mice, as male mice lacking β-endorphin gain more weight compared to their adult wild-type counterparts [58]. However, food reward was not assessed in the former study to compare to the latter study. Thus, several methodological factors may add to these discrepancies.

Variation in dietary protocols may also influence the extent to which β-endorphin signaling contributes to feeding behavior. Differences in dietary composition, duration of high-fat diet exposure, and prior experience with palatable foods can alter reward circuits and create distinct behavior outcomes. Studies have shown that chronic exposure to highly palatable diets induces neuroadaptations within opioid-sensitive reward pathways, potentially modifying the contribution of β-endorphin to subsequent feeding behavior [57,60,62]. Likewise, differences in feeding schedules or food restriction protocols may change the contributions of homeostatic and hedonic mechanisms, resulting in different behavioral phenotypes across studies. Consequently, the effects of β-endorphin deficiency may become more apparent under conditions of prolonged dietary exposure or heightened reward salience.

Moreover, interpretation of findings from constitutive knockout models should be approached with caution. Lifelong absence of β-endorphin may lead to developmental compensation by other neurochemical systems involved in feeding and reward regulation. Such compensatory adaptations could mask or alter the behavioral consequences of β-endorphin deficiency and may contribute to differences across observed studies. Additional biological variables, including mouse genetic background, age, sex, and behavioral testing procedures, may further influence experimental outcomes and complicate direct comparisons between studies. These factors emphasize the importance of considering methodological differences when interpreting conflicting findings in literature.

Notably, male β-endorphin-deficient mice have been reported to exhibit increased body weight relative to their wild-type controls [58], despite evidence of reduced food reward and, in some studies, reduced binge-eating [60]. This observation suggests that β-endorphins may influence metabolic regulation through mechanisms that extend beyond hedonic feeding. Consistent with this possibility, alterations in glucose homeostasis have also been reported following disruption of β-endorphin signaling (Tolentino and Lutfy, unpublished data). Therefore, differences in metabolic phenotype and feeding behavior may reflect distinct physiological functions of β-endorphin that are not always captured by individual behavioral paradigms. Future studies using inducible or region-specific knockout models may help distinguish the direct effects of β-endorphin signaling from developmental adaptations and further clarify its role in feeding and metabolism.

Taken together, the current evidence suggests that β-endorphins are most consistently involved in the hedonic and sensory aspects of feeding, particularly in response to palatable tastes and fats, rather than serving as primary determinants of overall food intake. Their effects appear highly context-dependent and are influenced by experimental design, dietary history, metabolic state, and compensatory adaptations. While β-endorphins contribute to food reward and reinforcement, current evidence does not support a singular or indispensable role for them in the development of compulsive eating. In contrast, enkephalins have been more strongly associated with modulating the motivational drive to obtain food. Together, these distinctions highlight the functional heterogeneity within the endogenous opioid system in regulating food reward. Indeed, we have recently reported that enkephalins may be involved in glucose homeostasis following the development of diet-induced obesity in mice [63] but not binge-eating or food reward [60].

#### 3.2.3. Nociceptin Signaling

Existing behavioral evidence suggests that nociceptin signaling may differentially regulate the motivational and hedonic aspects of feeding behavior. Mendez and colleagues found that intracerebroventricular (ICV) administration of nociceptin increased the number of licking bouts for sucrose and sucralose in wild-type mice, an effect which was absent in NOP knockout mice. These findings support that nociceptin signaling may regulate the initiation of hedonic food consumption, as increased number of licking bouts is associated with increased motivational behavior [64]. Although the mechanism by which nociceptin signaling affects food intake is not fully delineated, researchers have proposed that nociceptin may inhibit anorexigenic POMC neurons in the ARC [65,66].

Not only does exogenous nociceptin regulate food intake, but studies have shown that endogenous nociceptin may also be involved in this process. Farhang and colleagues showed that under fasting conditions, NOP knockouts showed reduced meal duration and portion sizes, compared to wild-type mice [65]. Additional pharmacological evidence also implicates nociceptin signaling in palatability-driven hyperphagia. Using an intermittent HFD access model, Hardaway and colleagues found that administration of a selective NOP antagonist, SB-612111 significantly reduced binge-like HFD intake in both male and female mice without altering their 24 h food intake. In contrast to fluoxetine, NOP antagonism selectively reduced palatable food consumption, suggesting that NOP signaling may preferentially modulate hedonic feeding rather than homeostatic food intake [19,67]. SB-612111 administration also reduced short-term consumption of HFD in male mice, which further reinforces the role of nociceptin in palatability-driven hyperphagia. Similarly, Statnick and colleagues found that another selective NOP antagonist, LY2940094 reduced fasting-induced feeding in mice, an effect that was not observed in NOP knockout mice. In separate rat models, LY2940094 attenuated overconsumption of palatable high-energy foods in lean rats and reduced body weight regain after a 30% caloric restriction. Collectively, Statnick’s rat and mice models support the idea that the reductions observed in feeding were attributed to NOP antagonism rather than other general, nonspecific effects [68].

Hardaway and colleagues also showed that nociceptin signaling contributes to hedonic feeding most prominently in the central amygdala. They identified a population of prepronociceptin-expressing neurons in the central amygdala (Pnoc^CeA^) that were activated during HFD consumption. Experiments conducted found that these neurons were necessary for both HFD intake and the development of HFD-induced increases in body weight and adiposity. Similar to findings of Hardaway and colleagues in 2016, chemogenetic inhibition of these neurons selectively reduced HFD consumption without significantly altering homeostatic chow feeding, suggesting nociceptin’s involvement in hedonic rather than homeostatic feeding. Interestingly, optogenetic activation of Pnoc^CeA^ neurons and their projections to the vBNST, PBN, and NTS produced reward-like behavior without stimulating palatable food intake. Together, these findings support a model where endogenous nociceptin signaling within the CeA contributes to the development of reward reinforcement of palatable foods, but is not the sole driver of hedonic feeding [55].

However, not all studies examining the relationship between nociceptin and food reward support its role in hedonic feeding. The function of nociceptin in feeding was examined by Koizumi and colleagues using NOP knockout mice to differentiate between its hedonic and homeostatic contributions to food intake. Under food restriction, NOP knockout mice showed reduced preference for a high-sucrose diet and lower high-fat diet intake under no-choice conditions. However, both effects disappeared entirely under ad libitum feeding, suggesting that the NOP’s influence on diet selection primarily depends on the homeostatic state rather than a stable change in palatability. In addition, conditioned place preference to high-fat diet under food-deprived conditions was largely unchanged, indicating that NOP does not play a primary role in mediating the rewarding or motivational properties of food. Instead, Koizumi and colleagues observed substantial disruptions in body weight, plasma leptin, glucose, and insulin in the NOP knockout mice. These results suggest that endogenous nociceptin may modulate diet selection as a part of homeostatic feeding, independent of the hedonic properties of food [69].

Taken together, within the endogenous opioid system, the nociceptin system plays a distinct role in feeding. Unlike β-endorphin-mediated amplification of hedonic value or enkephalin-mediated motivational drive, NOP receptor signaling appears to influence the preferential consumption of palatable foods under binge-like or high-fat feeding conditions through mechanisms that still are not completely understood. The finding that NOP antagonism reduces binge-like HFD intake without affecting homeostatic feeding suggests that the nociceptin system may be a potential pharmacological target for binge-eating disorder. However, further research is needed to clarify the contributions of hedonic and homeostatic processes to nociceptin’s role in feeding behavior.

#### 3.2.4. Dynorphin/Kappa Opioid Receptor (KOP) System

Though dynorphin binding to KOPs generally produces aversive behaviors in rodents and dysphoria in humans [15,16,17], studies have shown that dynorphin-like agonists and KOP activation increase feeding in both hungry and satiated rats, suggesting that KOP-mediated feeding occurs independently of energy state [70,71,72,73,74]. Likewise, KOP antagonists, such as norbinaltorphimine, reduce KOP-mediated feeding responses [75,76]. Several studies have shown that the medial hypothalamus and VTA appear to be the brain regions that mediate the orexigenic effects of dynorphin and other dynorphin-like peptides. Local injections of dynorphins into both the medial hypothalamus and VTA promote feeding [77,78]. Interestingly, Hamilton and Bozarth showed that dynorphin was 50,000 times more potent than morphine in stimulating food intake, reinforcing the idea that dynorphin signaling plays an important role in regulating feeding behavior [78]. However, despite numerous studies that support a role for dynorphin/KOP system in feeding regulation, the specific neural circuits and region-dependent mechanisms of these effects remain unclear. Likewise, less is known about the role of this system in food reward and compulsive binge eating. Thus, future studies need to be geared toward addressing the role of the dynorphin/KOP in food reward, binge eating, and compulsive eating.

### 3.3. Opioid Dysregulation and Compulsive Eating

#### 3.3.1. MOP Signaling and Binge-Eating Disorder

Dysregulation of MOP signaling has been increasingly deemed a key mechanism linking repeated exposure to palatable foods to compulsive eating behaviors observed in binge-eating disorder (BED) and obesity. Though endogenous opioids are important for normal reward processing, chronic stimulation of this system by palatable foods may lead to neuroadaptations that drive excessive food intake. Thus, rather than acting as primary drivers under normal baseline conditions, changes in MOP signaling may contribute to the development of compulsive eating behaviors. Indeed, we have shown that food reward in the place conditioning paradigm was blunted in mice lacking MOP with prior HFD exposure compared to their wild-type littermates [60]. In contrast, it did not differ between naïve mice lacking MOP and their wild-type littermates [79]. Together, these results suggest that a somewhat sensitized system develops involving MOP in mice with prior HFD exposure. Thus, MOP represents a promising target for developing new therapeutic treatments for compulsive eating behaviors.

Pharmacological evidence suggests that MOP signaling is more closely associated with the motivation for palatable foods than with the regulation of baseline energy needs. In their preclinical study, Ignar and colleagues showed that treatment with the selective MOP inverse agonist GSK1521498 reduced nocturnal feeding, lowered preference for sucrose, and decreased the effort animals would exert to obtain palatable food. Interestingly, the following effects were observed without eliminating feeding altogether, suggesting that MOP antagonism selectively reduces the reinforcing value of palatable foods rather than inducing nonspecific anorexia. These findings support that dysregulated MOP signaling may contribute to excessive hedonic feeding by enhancing the motivational salience of palatable foods [80].

Translational studies in humans further support a role for MOP signaling in the dysregulation of cue-driven feeding behavior. In individuals with binge-eating behaviors, GSK1521498 reduced attentional bias toward food cues in a dose-dependent manner, without impairing broader cognitive functions (e.g., working memory and sustained attention). By reducing attentional capture by food cues, MOP antagonists may weaken the cue-driven urges that are often attributed to the onset of binge episodes, suggesting that dysregulated MOP signaling contributes to heightened cue reactivity in compulsive eating [81].

Animal models have been particularly useful in clarifying how MOP antagonism affects the behavioral aspects of binge-eating. Direct comparisons between GSK1521498 and naltrexone reveal that while both compounds reduce binge-like hyperphagia, GSK1521498 more effectively suppresses anticipatory food seeking. This distinction is significant, as anticipatory responding reflects the learned and motivational components of binge-eating rather than consumption itself. These findings suggest that MOP dysregulation may preferentially contribute to the “wanting” component of feeding behavior rather than “liking,” aligning binge-eating more closely with addiction-like processes than with simple overeating [42,82].

Neuroimaging studies reinforce this dissociation between motivational drive and hedonic experience. In individuals with binge-eating tendencies, GSK1521498 reduced activation in reward-related regions, such as the pallidum and putamen, during exposure to high-calorie food cues and decreased the effort required to obtain these cues. However, participants continued to report similar or even increased liking of the foods. This dissociation between motivational drive and hedonic experience mirrors patterns observed in substance use disorders, where compulsive seeking persists despite reduced hedonic reward. Such findings suggest that opioid dysregulation in BED may bias behavior toward habitual, reward-seeking processes that are independent of hedonic pleasure [83].

Human PET imaging studies provide further evidence of MOP system alterations in binge-eating and obesity. Reduced MOP availability has been observed in both BED and morbid obesity, indicating either receptor downregulation due to chronic endogenous opioid release or a preexisting vulnerability within the opioid system. Notably, the lack of differences in MOP availability between BED and non-BED obese subjects suggests that opioid dysregulation is not specific to BED but instead may reflect a shared neurobiological consequence of repeated exposure to palatable foods. This raises important questions about causality and highlights the need for longitudinal studies to determine whether MOP alterations precede or follow disordered eating patterns [84].

Collectively, these findings suggest that MOP dysregulation does not simply increase food intake but instead reshapes the motivational and cognitive processes that regulate feeding behavior. Rather than directly driving hunger, altered opioid signaling appears to enhance cue responsivity, anticipatory reward, and compulsive food seeking, while leaving hedonic experience relatively intact. This dissociation has important implications for treatment, suggesting that effective interventions may need to target maladaptive motivational processes rather than hedonic enjoyment alone.

#### 3.3.2. Opioid-Dopamine Interactions

The reinforcing effects of palatable food arise from the close interactions between both the endogenous opioid system and mesolimbic dopaminergic circuits. DA signaling from the VTA to the NAc is primarily associated with motivation and reward anticipation [85]. In contrast, opioid peptides, particularly β-endorphins derived from POMC, enhance the pleasurable component of food consumption [86], particularly in animals with prior HFD exposure [60]. These systems operate in an integrated manner as shown by the direct influence of activated MOPs on dopaminergic neuronal activity, altering DA release across interconnected brain regions responsible for reward processing [87].

**Figure 3:**
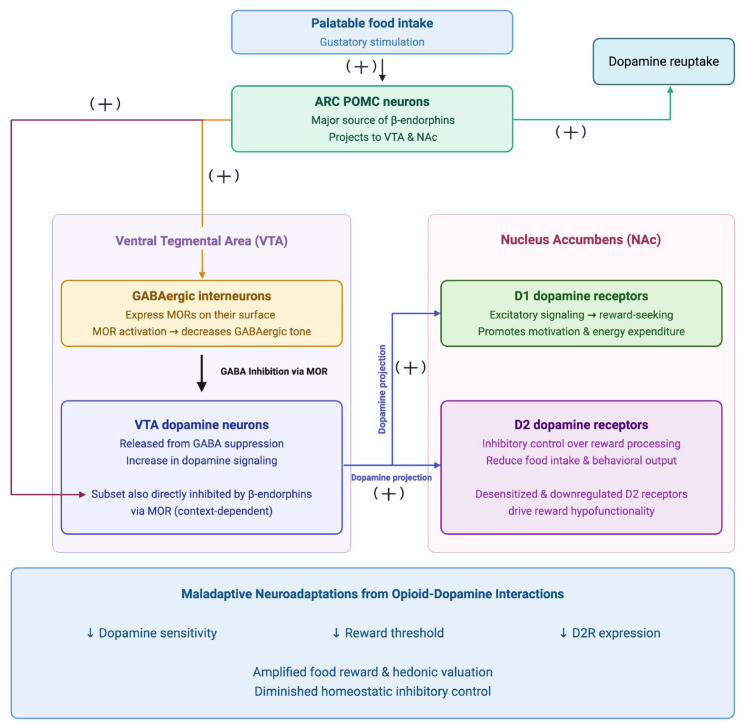
A simplified flowchart diagram of the impact of palatable food intake on dopamine signaling in the mesolimbic reward circuitry. Palatable food stimuli promote the release of β-endorphins by ARC POMC neurons, which project to the VTA. In the VTA, β-endorphins bind to MOPs expressed on GABAergic interneurons, resulting in MOP-receptor-mediated GABA inhibition. This results in the disinhibition of VTA dopamine neurons, which increases dopamine signaling to the NAc. However, a small subset of VTA dopamine neurons is inhibited directly by β-endorphins through MOPs, indicating that opioid regulation of dopamine neurons is projection-specific and bidirectional rather than solely excitatory. Nonetheless, the general increase in dopamine signaling is relayed to the NAc, where dopamine D1 activation promotes reward-seeking, excitatory signaling and energy expenditure, and dopamine D2 receptor activation provides inhibitory control over hedonic processing and food intake. With chronic stimulation from palatable food intake, D2 receptors become desensitized and downregulated, which contributes to reward hypofunctionality and diminished homeostatic inhibitory control. Abbreviations: ARC, arcuate nucleus; D2R, dopamine D2 receptor; GABA, gamma-aminobutyric acid; MOP, mu-opioid receptor; NAc, nucleus accumbens; POMC, proopiomelanocortin; VTA, ventral tegmental area.

Within the VTA, opioids influence DA neuron excitability through both direct and indirect mechanisms (Figure 3). MOPs are expressed on local GABA-ergic interneurons in addition to subsets of DA neurons. Acute MOP activation disinhibits DA neurons by suppressing GABAergic tone, thereby increasing DA release in downstream targets, such as the NAc [88]. However, recent optogenetic evidence has shown that the activation of ARC POMC neurons—major sources of β-endorphin—can directly inhibit specific VTA DA subpopulations via MOP signaling, indicating that opioid control of DA activity varies by projection and context [89,90]. Thus, this raises the possibility that endogenous opioid signaling plays a dual role in mesolimbic DA neurotransmission, either potentiating or suppressing it depending on the underlying circuitry and physiological states, including stress and metabolic imbalances. At the level of the NAc, opioids further regulate dopaminergic tone by modulating presynaptic dopaminergic dynamics. Earlier in vivo neurochemical studies suggest that β-endorphin alters striatal DA handling, enhancing DA reuptake in an opioid receptor-dependent manner [91]. The resulting DA activity in the NAc subsequently influences food reward and intake through interactions between palatable food stimuli, DA ligands, and different DA receptor subtypes within the mesolimbic reward circuitry.

DA release in the NAc encodes multiple, dissociable aspects of food-directed behavior rather than a single motivational signal. Phasic DA release, occurring on a sub-second timescale, is thought to encode a reward prediction error, the mismatch between expected and received reward, and functions as a teaching signal that updates the learned value of food-associated cues [92,93]. Tonic DA levels fluctuate over a slower timescale and appear to track a more sustained representation of relative reward value that shapes ongoing motivation and effort allocation. Fiber photometry and voltammetry studies have dissociated these cue-evoked phasic transients from slower extracellular DA fluctuations, suggesting they carry at least partially separable information [92]. Both signals converge on D1 and D2 receptor-expressing medium spiny neurons (MSN) in the NAc. However, the classical framework in which D1 activation is uniformly excitatory and reward-promoting while D2 activation is uniformly inhibitory and aversive oversimplifies basal ganglia circuitry. Unlike in the dorsal striatum, D1- and D2-MSNs in the NAc are not strictly segregated by projection target, and both populations can drive reward or aversion depending on the temporal pattern of their activity rather than on receptor identity alone [94,95]. D2 receptor signaling is also regionally heterogeneous: D2 receptor-mediated currents show slower kinetics and higher dopamine sensitivity in the NAc shell than in the dorsal striatum, a regional difference that is itself altered by chronic drug exposure and may be similarly susceptible to chronic palatable food exposure [96]. Considered together, these findings suggest that the reward hypofunctionality attributed to D2 receptor downregulation after repeated palatable food exposure is one component of a broader, dynamic, cell type- and circuit-specific pattern of dopaminergic adaptation, rather than a simple loss of a single inhibitory brake on feeding. Thus, dysregulated DA signaling, especially in cases of excessive DA transmission, can disrupt normal hedonic processes. Upon repeated exposure to elevated DA signaling and palatable foods, D2 receptors are downregulated and desensitized, leading to reward hypofunctionality and overconsumption to achieve the same hedonic effects [97,98]. Furthermore, dysregulated opioid-DA coupling may potentially amplify the hedonic valuation of energy-dense foods while progressively diminishing homeostatic inhibitory control [99]. Interestingly, we have recently discovered that the value of regular diet decreases following HFD exposure and this response is attenuated in mice lacking MOP [60]. However, further research is needed to delineate the underlying mechanisms of this alteration and identify the neuroanatomical site or the pathway involved in this process.

Taken together, chronic overstimulation of MOPs within mesolimbic hedonic circuits can lead to maladaptive dopaminergic adaptations, including but not limited to, alterations in DA sensitivity, lower reward thresholds, and excessive intake to achieve equivalent hedonic effects [100]. Consequently, dysregulated opioid modulation of dopaminergic pathways links palatable food exposure to compulsive eating phenotypes, presenting opioid-DA interactions as a critical component for the etiology of obesity and binge-eating. Indeed, a combination of naltrexone and bupropion (CONTRAVE^®^) has been developed for the treatment of obesity [101].

## 4. Conclusions

The evidence reviewed highlights the importance of the endogenous opioid system in hedonic feeding and binge-eating. The role of individual opioid peptides is different but complementary. β-endorphins modulate the hedonic value of food in a context-dependent manner, whereas enkephalins regulate the motivational drive to obtain it. Furthermore, nociceptin plays a distinct role within the context of feeding. Unlike β-endorphins or enkephalins that modulate hedonic value and motivational drive of food, respectively, NOP receptor signaling selectively promotes palatable food intake under binge-like conditions. However, NOP antagonism reduces HFD intake without disrupting homeostatic feeding. This distinction makes NOPs a potential pharmacological target for BED specifically.

In addition, dysregulated MOP signaling appears to reshape the cognitive and hedonic processes that govern feeding behavior, contributing to maladaptive reward processing, increased motivation for palatable foods, and impaired inhibitory control over intake. These effects are further amplified by downstream dopaminergic signaling, including D2 receptor downregulation and reward hypofunctionality, which lowers the threshold for compulsive eating while weakening homeostatic inhibitory control. Together, these neuroadaptations parallel the mechanisms observed in substance use disorders, reinforcing the idea that binge-eating is, at least in part, a reward-driven condition associated with compulsive, habitual processes and overconsumption. Current pharmacological interventions targeting opioid signaling, particularly MOP antagonists such as naltrexone [82] and its combination with bupropion (CONTRAVE^®^), have shown promise in reducing food craving, binge-like consumption, and attentional bias toward palatable foods [101].

Despite these preclinical and early clinical signals, the real-world clinical efficacy of opioid-targeted pharmacotherapy for obesity and BED has generally been modest and variable across patient populations. Naltrexone produces minimal weight loss on its own, and even the FDA-approved naltrexone/bupropion combination (CONTRAVE^®^) yields placebo-corrected weight loss of roughly 5%, with adverse effects such as nausea, constipation, and headache contributing to discontinuation rates that limit long-term adherence [102]. GSK1521498 has shown encouraging effects on food-cue reactivity and attentional bias in early-phase trials but has not advanced to FDA approval. Its clinical development has been constrained by two factors: questions of receptor selectivity relative to naltrexone, and considerable inter-individual variability in response. This variability may partly reflect genetic differences in opioid receptor signaling [79,81]. This variability, combined with the clinical and biological heterogeneity of obesity and BED, likely reflecting multiple distinct underlying neurobiological subtypes, may help explain why single-target opioid therapies have not consistently reproduced the effect sizes observed in preclinical models. Precision medicine approaches that stratify patients using genetic, neuroimaging, or behavioral biomarkers of opioid system function prior to treatment, together with combination pharmacotherapy pairing opioid receptor modulation with agents acting on complementary pathways, represent promising strategies for improving on this variability.

Among these complementary pathways, glucagon-like peptide-1 (GLP-1) receptor agonists have substantially reshaped the clinical landscape of obesity treatment. Semaglutide and tirzepatide produce considerably greater weight loss than naltrexone/bupropion, and their actions extend well beyond peripheral appetite suppression: GLP-1 receptors are expressed within mesolimbic circuitry, and GLP-1 receptor agonists reduce food-seeking behavior, dampen motivation for palatable food, and modulate dopaminergic signaling within the VTA and NAc [103,104]. In a direct clinical comparison, semaglutide reduced binge-eating episodes more effectively than the currently prescribed BED medications lisdexamfetamine and topiramate [105]. Mechanistically, GLP-1 receptor agonism and opioid receptor antagonism appear to converge on overlapping mesolimbic circuitry through distinct molecular routes: GLP-1 signaling appears to dampen dopaminergic drive upstream in the reward cascade, while MOP antagonism blunts the hedonic amplification of that signal downstream. This raises the possibility that combined or sequential use of these drug classes could address both the motivational and hedonic components of compulsive eating more comprehensively than either strategy alone. Direct trials testing opioid–GLP-1 combination therapy specifically for BED remain lacking, however, and this represents an important direction for future translational research.

However, this review has several limitations. As a narrative review, the studies we chose to incorporate may have introduced some selection bias. In addition, the emphasis on preclinical versus human studies may not fully reflect the translational complexity of these pathways in humans, as few clinical studies have focused on the aforementioned opioid- and dopamine-related pathways. For example, current treatments remain limited by variability in efficacy, side effects associated with non-compliance, and an incomplete understanding of long-term outcomes, especially in humans. Although GSK1521498 is not yet FDA-approved for clinical use, it is currently being investigated in various clinical studies in the context of addictive behaviors associated with food and alcohol use. Future therapeutic strategies may benefit from combining opioid-based pharmacotherapy with behavioral interventions and targeting specific neural pathways involved in reward and inhibitory control. The neurobiological changes associated with the pathophysiology of food reward and compulsive eating occur alongside a wide range of other neuropeptide systems, including but not limited to hypothalamic feeding circuits, cortical inhibitory circuits, and hormones involved in feeding and reward that act centrally such as GLP-1. As stated above, GLP-1s such as semaglutide are more effective and act on overlapping brain circuitry, suggesting that combination therapy of opioid antagonist with GLP-1s is a potentially promising, but still untested, intervention for food reward and compulsive eating. Achieving this, however, depends on addressing key gaps in our understanding of the opioid system. For example, the characterization of the relative contributions of hedonic and homeostatic mechanisms to nociceptin’s effect is still not completely understood. Human data on NOP receptor signaling in BED is also largely lacking, compared to other opioid peptides and receptors. Similarly, whether MOP alterations in binge-eating disorder and obesity reflect a preexisting condition or a consequence of repeated palatable food exposure remains unclear. This distinction has significant implications for identifying at-risk individuals and determining when pharmacological interventions are most effective. Further research is therefore needed to clarify this question alongside peptide-specific roles and the long-term neurobiological consequences of chronic palatable food exposure. Integrating opioid signaling with dopaminergic and metabolic pathways may provide a more comprehensive framework for treatment, allowing for the development of more effective, mechanism-based interventions for obesity and binge-eating disorder.

In addition, sex differences are an important yet often overlooked aspect of opioid-mediated signaling and food reward. The findings from existing preclinical studies indicate that biological sex appears to influence sensitivity to the rewarding and reinforcing properties of opioids. For example, male rats appear to be less sensitive than female rats to these effects, with lower opioid intake and slower acquisition time for oral and intravenous opioid self-administration [106,107,108,109]. On the other hand, male rats also show morphine-induced conditioned place preference within a smaller dose range compared to female rats [110,111]. However, this difference depends on a variety of factors, including but not limited to reinforcement schedule, dosage, and level of food restriction [112]. In addition, sex differences can also be specific to certain receptor subtypes. Craft and colleagues have shown that males exhibit greater sensitivity than females to the discriminative stimulus effect of KOP but not MOP opioid agonists [113,114,115]. Furthermore, gonadal hormones also appear to play a role in dopamine signaling. Kokane and Perrotti have shown that estradiol influences dopamine activity within the mesolimbic reward system by enhancing rewarding and reinforcing behaviors mediated by opioid drugs [116]. Taken together, these sex differences are likely to affect findings related to opioid-mediated feeding and food reward. Yet, this aspect remains largely unexplored and represents an important direction for future studies. More specifically, sex differences should be considered as a factor when conducting studies of opioid- and dopamine-related mechanisms underlying food reward and binge-eating.

## Data Availability

No new data were created or analyzed in this study.

## Abbreviations

The following abbreviations are used in this manuscript:

AgRP	Agouti-related peptide
ARC	Arcuate nucleus
BED	Binge-eating disorder
CART	Cocaine- and amphetamine-regulated transcript
CeA	Central amygdala
D1	Dopamine D1 receptor
D2/D2R	Dopamine D2 receptor
DA	Dopamine
DOP	Delta-opioid receptor
GABA	γ-aminobutyric acid
GLP-1	Glucagon-like peptide-1
HFD	High-fat diet
ICV	Intracerebroventricular
KOP	Kappa-opioid receptor
LH	Lateral hypothalamus
MOP	Mu-opioid receptor
MSN	Medium spiny neurons
NAc	Nucleus accumbens
NOP	Nociceptin opioid peptide receptor
NPY	Neuropeptide Y
NTS	Nucleus of the solitary tract
PBN	Parabrachial nucleus
PET	Positron emission tomography
Pnoc^CeA^	Prepronociceptin-expressing neurons of the central amygdala
POMC	Proopiomelanocortin
PVN	Paraventricular nucleus
vBNST	Ventral bed nucleus of the stria terminalis
VTA	Ventral tegmental area
